# Male frequency in *Caenorhabditis elegans* increases in response to chronic irradiation

**DOI:** 10.1111/eva.13420

**Published:** 2022-09-02

**Authors:** Loïc Quevarec, Denis Réale, Elizabeth Dufourcq‐Sekatcheff, Clément Car, Olivier Armant, Nicolas Dubourg, Christelle Adam‐Guillermin, Jean‐Marc Bonzom

**Affiliations:** ^1^ PSE‐ENV/SRTE/LECO, Cadarache Institut de Radioprotection et de Sûreté Nucléaire (IRSN) Saint Paul Lez Durance France; ^2^ Département des Sciences Biologiques Université du Québec à Montréal Montréal Quebec Canada; ^3^ PSE‐SANTE/SDOS/LMDN, Cadarache Institut de Radioprotection et de Sûreté Nucléaire (IRSN) Saint Paul Lez Durance France

**Keywords:** *Caenorhabditis elegans*, experimental evolution, selection, sex ratio, sexual conflict, stressful environment

## Abstract

Outcrossing can be advantageous in a changing environment because it promotes the purge of deleterious mutations and increases the genetic diversity within a population, which may improve population persistence and evolutionary potential. Some species may, therefore, switch their reproductive mode from inbreeding to outcrossing when under environmental stress. This switch may have consequences on the demographic dynamics and evolutionary trajectory of populations. For example, it may directly influence the sex ratio of a population. However, much remains to be discovered about the mechanisms and evolutionary implications of sex ratio changes in a population in response to environmental stress. Populations of the androdioecious nematode *Caenorhabditis elegans*, are composed of selfing hermaphrodites and rare males. Here, we investigate the changes in the sex ratio of *C. elegans* populations exposed to radioactive pollution for 60 days or around 20 generations. We experimentally exposed populations to three levels of ionizing radiation (i.e., 0, 1.4, and 50 mGy.h^−1^). We then performed reciprocal transplant experiments to evaluate genetic divergence between populations submitted to different treatments. Finally, we used a mathematical model to examine the evolutionary mechanisms that could be responsible for the change in sex ratio. Our results showed an increase in male frequency in irradiated populations, and this effect increased with the dose rate. The model showed that an increase in male fertilization success or a decrease in hermaphrodite self‐fertilization could explain this increase in the frequency of males. Moreover, males persisted in populations after transplant back into the control conditions. These results suggested selection favoring outcrossing under irradiation conditions. This study shows that ionizing radiation can sustainably alter the reproductive strategy of a population, likely impacting its long‐term evolutionary history. This study highlights the need to evaluate the impact of pollutants on the reproductive strategies of populations when assessing the ecological risks.

## 1 INTRODUCTION

Outcrossing, the fusion of gametes from distinct individuals, plays an important role in evolution (Otto & Lenormand, 2002), is widespread in multicellular organisms, and confers many advantages. For example, the recombination caused by genetic crossbreeding generates offspring that are genetically different from their parents, and both theoretical and empirical studies have shown that the resulting increase in genetic diversity improves population persistence and evolutionary potential in response to novel environmental stresses or conditions (Colegrave, 2002; Morran et al., 2009a; Seudre et al., 2018). Genetic recombination also facilitates the elimination of deleterious mutations (Crow, 1994) and spreads beneficial mutations (Otto, 2009). In contrast, asexual or obligatory self‐fertilizing species accumulate deleterious mutations through the Muller ratchet mechanism (Felsenstein, 1974; Morran et al., 2009a; Muller, 1932) and homozygosity (Glémin & Galtier, 2012). These processes lead to the expression of recessive mutations and inbreeding depression and may lower the evolutionary potential of the population (Archetti, 2004). Although the subject is still strongly debated, asexual or obligatory self‐fertilizing species are expected to have higher extinction rates than outcrossing species (Glémin et al., 2019; Goldberg et al., 2010; Ho & Agrawal, 2017). However, these modes of reproduction can provide fitness gains. For example, they can maintain beneficial gene combinations that would be easily broken by outcrossing (i.e., outbreeding depression) (Dolgin et al., 2007) and improve the chance of reproduction at low population density (Morran et al., 2009b; Teotónio et al., 2012).

Outcrossing may, therefore, be particularly effective in a changing environment (Colegrave, 2002; Morran et al., 2009a). This hypothesis is supported by the finding that environmental stresses promote sexual reproduction in some organisms, such as daphnia or nematodes that usually breed asexually (Alonzo et al., 2008; Camp et al., 2019; Dacks & Roger, 1999; Gemmill et al., 1997). Outcrossing, however, also leads to some significant costs (Lehtonen et al., 2012; Otto, 2009), such as reduced growth rate (Gibson et al., 2017; Smith & Maynard‐Smith, 1978), increased time and energy spent finding mates, increased disease transmission, and a higher vulnerability to predators during mating (Grosmaire, 2018; Otto & Lenormand, 2002). Whether a population uses asexual reproduction to avoid these costs or sexual reproduction to benefit from outcrossing will, therefore, depend on many factors.

Outcrossing requires the presence of both male and female reproductive functions. The sex ratio influences the sexual selection and is an important parameter in the evolution of a species (Janicke & Morrow, 2018). It also plays an essential role in the demography, mating system, and genetics of a population, and can influence its evolutionary trajectory (Freedberg & Taylor, 2007; Hartl et al., 1997; Rood & Freedberg, 2016; Sowersby et al., 2020). According to Fisher's principle (Fisher, 1930), frequency‐dependent selection maintains a balanced sex ratio in most populations. However, the sex ratio varies widely between gonochoric taxa, and sex can be determined genetically or plastically in response to environmental signals (Székely et al., 2014; West & Sheldon, 2002). Under stressful conditions, selection may favor one sex if that sex provides a greater fitness than the other. For example, maternal stress alters the relative cost of producing each sex (Geffroy & Douhard, 2019), hence production of the lowest cost sex could confer a selective advantage (Myers, 1978). For example, Agaonid fig wasps, *Ceratosolen galili*, adjust their sex ratio in response to the number of foundresses laying eggs in a fig, which presumably functions to limit local mate competition in the offspring (Greeff et al., 2020). Thus, the sex ratio of a population may change under new selection pressures (Sowersby et al., 2020) and can, in turn, affect the evolution of genetic sex‐determining mechanisms (Mank et al., 2006). However, much remains to be discovered about the mechanisms and evolutionary implications of sex ratio changes in a population in response to environmental stress (Sowersby et al., 2020).

At certain doses, ionizing radiation is a powerful environmental stressor, and its emission in the environment has increased with human activity since the end of World War II (Rhodes et al., 2020). For instance, the accidents at the Chernobyl and Fukushima nuclear power plants have released 1.4 × 10^19^ Bq (IAEA, 2006) and 5.2 × 10^17^ Bq (Okano et al., 2016) of radionuclides into the environment, respectively. Following these accidents, many surrounding ecosystems have been contaminated, with negative effects of radiocontamination on wildlife (Aliyu et al., 2015; Cannon & Kiang, 2020; Geras'kin et al., 2008; Møller & Mousseau, 2006). Ionizing radiation can have detrimental effects on the reproduction of mammals and fish, such as delayed reproduction or a decrease in the weight and survival of embryos (Real et al., 2004) and has also been found to have deleterious effects on reproduction in many invertebrate species (Dallas et al., 2012; e.g., *Caenorhabditis elegans* Buisset‐Goussen et al., 2014; Dubois et al., 2018; Lecomte‐Pradines et al., 2017; Maremonti et al., 2019). Irradiation may also affect the sex ratio of populations, though more work is needed in this area. In humans, the effect of ionizing radiation on the sex ratio has been studied extensively, without consistent evidence of a significant impact (Terrell et al., 2011). In contrast, a change in the sex ratio induced by internal alpha irradiation, caused mainly by an accumulation of ^241^Am in the tissues, has been observed in *Daphnia magna* exposed over three generations, with nonreproducing males appearing at the F2 generation and accounting for 31% of the total daphnids (Alonzo et al., 2008). In the Chernobyl Exclusion Zone, female barn swallows (*Hirundo rustica*) showed higher mortality than males, and this supposedly affected the sex ratio of the population (Møller et al., 2012). To the best of our knowledge, the effects of external exposure to ionizing radiation on sex ratio dynamics have never been studied.

In this article, we studied the sex ratio of a population of *C. elegans* experimentally exposed to gamma radiation for 60 days or around 20 generations. The aim of this study was (1) to test whether exposure to gamma radiation changes the sex ratio of *C. elegans* populations and (2) to investigate the mechanisms underlying these changes. The metazoan *C. elegans* (Nematoda, Rhabditidae) is an androdioecious organism. *C. elegans*, and more broadly the genus *Caenorhabditis*, is particularly useful for studying the evolution of sexual interactions and sexual conflict (Palopoli et al., 2015). It is also ideal for investigating evolutionary questions about outcrossing and sex ratio because populations are mostly composed of hermaphrodites XX with a few rare males XØ (Herman, 2005). Hermaphrodites reproduce mainly by self‐fertilization, and their limited sperm quantity restrains their offspring production to about 300 individuals, all of which will be hermaphrodites (Barr et al., 2018; Chasnov, 2013; Cutter et al., 2019). However, hermaphrodites can also mate with males, and mating with several males can produce up to about 1000 offspring because male sperm is produced continually and in large numbers (Chasnov, 2013; Cutter et al., 2019; Singson, 2001). In addition, sperm competition induced by the presence of males could increase hermaphrodite sperm counts (Cutter, 2004). Oocytes sired by a male produce offspring that are 50% male and 50% hermaphrodite.

Male *C. elegans* are rare in the wild, between 0 and 22% of the population, and their frequency always remains lower than that of hermaphrodites (Anderson et al., 2010). The relative rarity of males suggests a selective advantage of self‐fertilization over outcrossing (Cutter et al., 2019; Stewart & Phillips, 2002), and this difference in reproductive strategies between the sexes can lead to fitness conflicts (Chapman, 2006). Indeed, it appears that mating with males can incur a cost, for example, increased mortality and decreased reproductive success of hermaphrodites who mate with males repeatedly (Cutter et al., 2019). However, male frequency strongly increases under severe stress (Lopes et al., 2008; Lynch et al., 2018; Morran et al., 2009a; Rose & Baillie, 1979). This observation suggests that there is either a decrease in the cost of outcrossing for hermaphrodites or an advantage to males under stressful conditions. Although the benefit of two‐parent reproduction is still a subject of debate in evolutionary biology, it has been suggested that in *C. elegans*, outcrossing enhances natural selection, allowing for faster adaptation and more effective purging of deleterious mutations (Cutter et al., 2019). Given these benefits to outcrossing in a stressful environment, we hypothesized that the selection for outcrossing would occur in the irradiated environments, resulting in an increase in the number of males. Mechanistically, since ionizing radiation causes a decrease in spermatid production (Maremonti et al., 2019), that affects the sperm limited hermaphrodites more strongly than males, we hypothesized that ionizing radiation would modify the sex ratio through a decrease in mating success of hermaphrodites who self‐fertilize compared to males and the consequential increase in production of male offspring. Oxidative stress and DNA damage induced by ionizing radiation decrease sperm quality and quantity by altering meiosis, decreasing sperm activation and viability and inducing germ‐cell apoptosis (Guédon et al., 2021; Maremonti et al., 2019).

## 2 MATERIAL AND METHODS

### 2.1 Test organism and population maintenance

We used the A6140 *C. elegans* population, created from a mixture of 16 wild isolates (Noble et al., 2017; Teotónio et al., 2012). This population had a large genetic diversity and contained about 20% males. We placed samples of the population on 6‐cm Petri dishes with 12 ml of nematode growth medium. Petri dishes were seeded with *Escherichia coli* bacteria (OP50 strain) ad libitum and exposed to UV radiation (Bio‐Link Crosslinker, λ = 254 nm; intensity = 200 μwatt.m^−2^) for 15 min to stop bacterial growth and to avoid uncontrolled heterogeneity in food availability. Nematode populations were cultured at 20°C and 80% relative humidity to have a generation time of approximately 3 days (Byerly et al., 1976). Before exposure, the stock population was maintained for at least 75 days or around 25 generations, in pairs of Petri dishes (referred to as A and B), with 500 individuals in each dish. Every 3 days, we transferred nematodes into new dishes to ensure they were fed ad libitum. To do so, we washed nematodes off the two Petri dishes with an M9 solution. We then pooled them in a 15 ml tube Falcon®, homogenized, and estimated the number of individuals based on six sample drops of 5 μl (Teotónio et al., 2012). Two separate volumes corresponding to 500 individuals at all developmental stages were then transferred into two new Petri dishes.

### 2.2 Irradiation conditions

**FIGURE 1:**
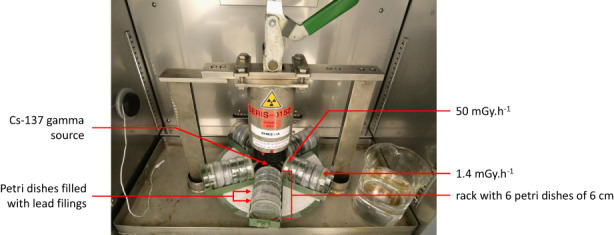
Irradiation system inside the incubator (credit: L. Quevarec/IRSN)

The external gamma radiation exposure was conducted at the Mini Irradiator for Radio Ecology ^137^Cs irradiation facilities, at the Institut de Radioprotection et de Sûreté Nucléaire (MIRE, IRSN; Figure 1). We used the same irradiation facilities, and the same protocol as previously described by Buisset‐Goussen et al. (2014), but with the following differences. The irradiators were placed in incubators with the controlled temperature at 20°C and 80% relative humidity. The populations of *C. elegans* were exposed to three dose rate gamma radiation treatments (five replicates per treatment): 0 (control treatment), 1.4 (low irradiation treatment), and 50 (high irradiation treatment) mGy.h^−1^. Low irradiation treatment had an environmental reality, Garnier‐Laplace et al. (2013) indicated that terrestrial wildlife could be exposed to dose rates up to ∼10 mGy.h^−1^ in the Chernobyl Exclusion Zone. High irradiation treatment was chosen because several studies have shown an impact on reproduction in *C. elegans* at a similar dose rate (Buisset‐Goussen et al., 2014; Dubois et al., 2018; Dufourcq‐Sekatcheff et al., 2021; Guédon et al., 2021; Maremonti et al., 2019). The dose rates were measured with radiophotoluminescence (RPL) microdosimeters twice during the experiment. As explained in Buisset‐Goussen et al. (2014), the Petri dishes were placed vertically in the irradiator to homogenize the dose received over the entire dish. To obtain the required dose rates, the plates were placed at different distances from the source and separated by shields (Petri dish filled with lead filings). For technical reasons, we placed the Petri dishes of the control condition in an identical incubator, with for only difference the absence of an irradiation system in the incubator.

### 2.3 Multigenerational experiment

**FIGURE 2:**
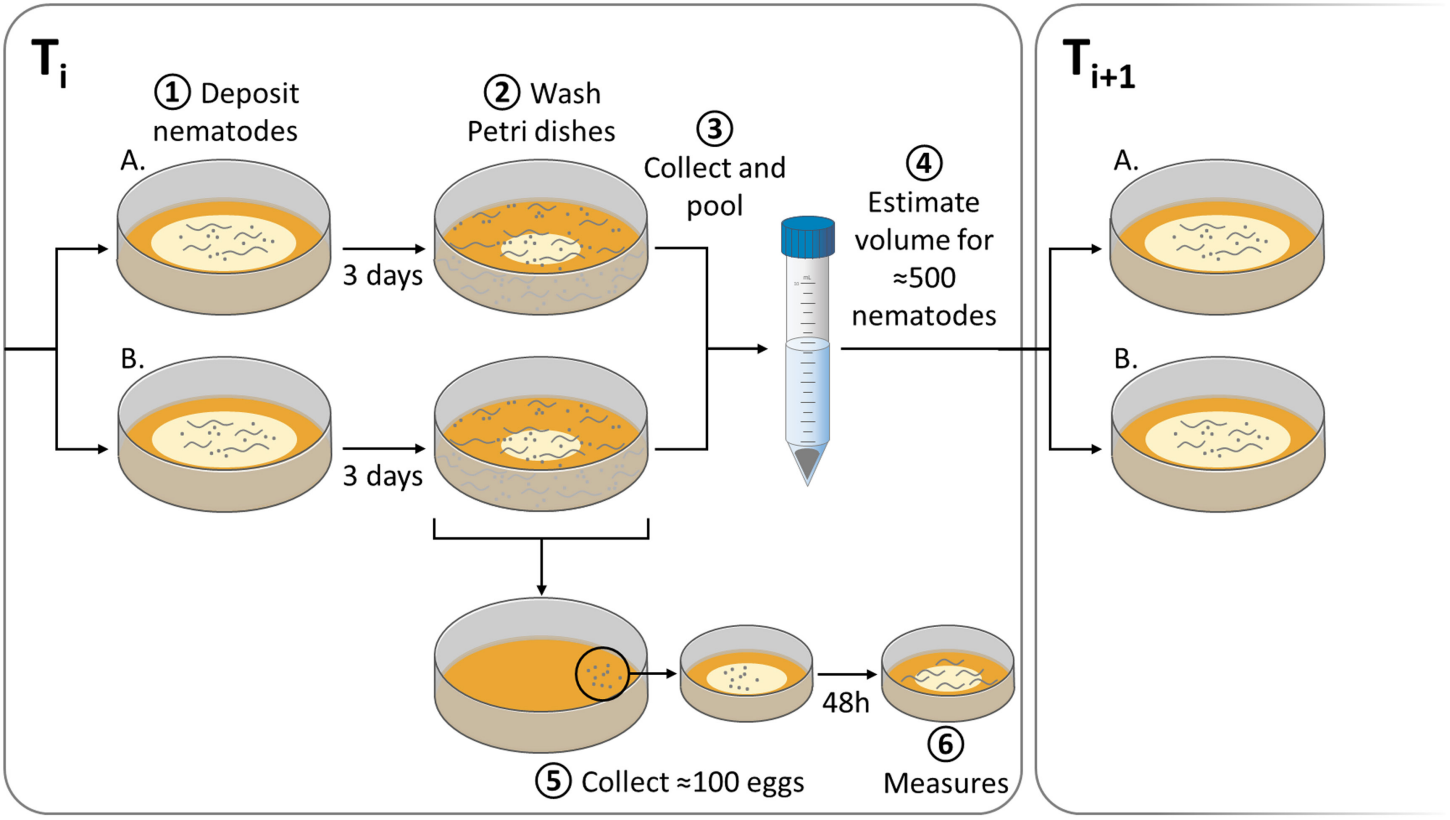
Schematic overview of the multigenerational experiment protocol for *C. elegans* populations under different gamma radiation treatments. For each treatment (0, 1.4, and 50 mGy.h^−1^), five replicates composed of two Petri dishes (which we will call A and B) were made. **1.** These plates were seeded with about 500 individuals of all stages (egg to adult). At the time of transfer (3 days), the two plates (A and B) were **2.** washed with M9 solution and **3.** pooled in a 15‐ml tube. **4.** A volume equivalent to ~500 nematodes was then used to seed two Petri dishes for the next transfer. This allowed us to have a population of about 1000 individuals per replicate at the beginning of each transfer, which were then spread over two Petri dishes. **5.** Simultaneously, ~100 eggs were collected and used to **6.** estimate the sex ratio for each replicate 48 h later

A multigenerational experiment was performed over 60 days (about 20 generations) with the same conditions for all populations except for the irradiation treatment (Figure 2). Dutilleul et al. (2014) found that this duration was long enough to detect plastic or evolutionary changes in *C. elegans*. We transferred samples of populations into new Petri dishes every 3 days. Although 3 days grossly correspond to a generation in natural conditions, gamma radiation delays growth and reproduction at high dose rates (Lecomte‐Pradines et al., 2017), and we cannot guarantee that every transfer corresponds to a generation. We thus describe the dynamics of changes during the experiment as a function of the number of 3‐day transfers.

We estimated the frequency of males relative to hermaphrodites at transfer 0, 2, 5, 8, 11, 14, 17, and 20. For each estimation, we transferred 100 eggs per replicate into new Petri dishes (3 cm) containing the same medium. Forty‐eight hours after the transfer, we counted males and hermaphrodites (L4 and young adult) with a stereomicroscope (Olympus SZX12, 1.6 × 90 magnification).

### 2.4 Reciprocal‐transplant experiments

**FIGURE 3:**
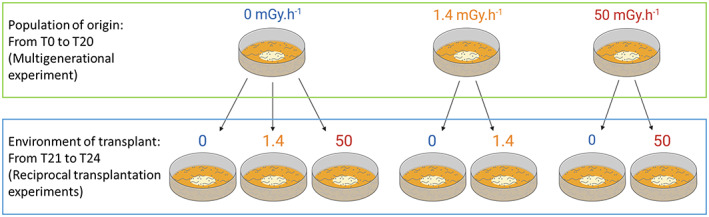
Schematic overview of the reciprocal‐transplant experiment design for *C. elegans* populations in three gamma radiation treatments. After 20 transfers in the initial environment (population of origin) (0, 1.4, and 50 mGy.h^−1^) during the multigenerational experiment, populations were placed in a second environment (environment of transplant) for four transfers. This partial reciprocal transplant was performed as shown here. The measurements of sex ratio were made after four transfers to ensure that the differences between populations were due to genetic differentiation and not parental effects

To test whether sex ratio changes were maintained if the stressor was removed, we performed a reciprocal transplant experiment between the control and both the low and the high irradiation treatment. For this experiment, we referred to the population that had evolved either in a controlled environment or in the two irradiated treatments as the “population of origin”, and the environment they had evolved in as the “environment of origin”. We referred to the novel environment; the populations were transferred to as the “environment of transplant” (Figure 3). At transfer 20, we collected 500 individuals from each population of origin and placed them either in the same environment (environment of origin) or in one of the two environments of transplant. We created five replicates for each condition of reciprocal transplant, resulting in 40 replicates in total. We then maintained these populations in the same conditions for 12 days or four transfers.

At the end of the fourth transfer of reciprocal transplants, we estimated the frequency of males relative to hermaphrodites. We measured the sex ratio after four transfers to ensure that the differences between populations were not due to parental effects (Badyaev & Uller, 2009; Dutilleul et al., 2014; Kawecki et al., 2012).

### 2.5 Stewart and Phillips model (SP model)

To investigate which factors could lead to a change in the sex ratio during the experiment, we modified Stewart and Phillips (2002) model of the evolution of androdioecy in *C. elegans*, following the equation:

$$m^{\prime }=\left(1-\sigma \right)\left(1+2u\right)\mathrm{\alpha m}/2T+\beta \left(1-\delta \right)\left(1-\mathrm{\alpha m}\right)u/T,$$

and:

$$T=\mathrm{\alpha m}\left[1-\sigma \left(1/2+u\right)\right]+\beta \left(1-\delta \right)\left(1-\mathrm{\sigma u}\right)\left(1-\mathrm{\alpha m}\right),$$

where α is the fertilization success of males, β is the proportion of eggs not fertilized by males that are self‐fertilized, *u* is the rate of nondisjunction of the X chromosome, δ is the degree of inbreeding depression in self‐fertilized offspring, σ is the relative viability difference between males and hermaphrodites, *m* is the male frequency in the current generation, and *m’* is the male frequency in the next generation. Following Kim (1985), results we predicted that ionizing radiation increases the rate of nondisjunction of the X chromosome (*u*), which should increase the bias in sex ratio in favor of males. Additionally, we predicted that the deleterious impact of radiation on spermatid production in hermaphrodites (Maremonti et al., 2019) decreases the proportion of self‐fertilized eggs β and increases the fertilization success of males α, which continuously produce sperm in large numbers (Cutter et al., 2019). This also should increase the proportion of males in the population.

Male fertilization success (α) covers many traits, such as the ability to find hermaphrodites or to mate with them. In the same way, the proportion of eggs not fertilized by male that are self‐fertilized (β) depends on trait such as the production of hermaphrodite sperm, the proportion of viable sperm, or the ability of male sperm to outcompete hermaphrodite sperm. Our goal, however, was not to analyze the exact role of these traits in these two variables, but rather to test whether the variables in Stewart and Phillips models (2002) could explain the temporal change in sex ratio.

The model analyzes the effect of generations, and we thus use the term generation to reflect the effect of time on sex ratio. Note that it differs from the experiment where we used the term transfer instead of generation, as explained above. However, it is important to remember that the dynamics of sex ratio in both the experiment and the model depend on across generation change. To model changes in male frequency over multiple generations, we created iterations where at each generation, we replaced the male frequency by the frequency of the previous generation. The model was also modified to incorporate changes in the α and β over generations. We empirically determined the baseline values of α and β by identifying the value at which the frequency of males was stable at 0.25, the level observed in the control condition. Thus, compared to the initial model, α = 0.65 and β = 0.15, *u* of the A6140 was fixed at 0.005 (Teotónio et al., 2012). Inbreeding depression is considered negligible in *C. elegans* (Stewart & Phillips, 2002). However, Teotónio et al. (2012) considered that inbreeding depression was present in diversified populations of 1000 individuals, but that it played a minor role in the male maintenance relative to adaptation. In our case, we had 500 individuals in each Petri dish (1000 per replicate population); we estimated that inbreeding depression had a weak contribution to male maintenance. Furthermore, with our experimental approach, we could not quantify inbreeding depression. We, thus, chose to focus on the parameters described above. δ was set to 0. In addition, σ was also set to 0 because at the highest dose rate (i.e., 50 mGy.h^−1^) used in this study, no mortality was expected (Clejan et al., 2006).

### 2.6 Statistical analysis

We used two statistical models to analyze the change in the sex ratio over the 20 transfers (data in Table S1). First, we used a Generalized Linear Mixed Model (GLMM) with R software (R Core Team, 2013) and the Ade4 package (Bougeard & Dray, 2018; Dray & Dufour, 2007) to examine the strong increase in male frequency observed over the first two transfers. In this model, the sex ratio corresponded to the frequency of males (i.e., number of males divided by the number of total individuals) and had a binomial distribution and a logit link function. No overdispersion of data was observed. The sex ratio was analyzed as a function of transfer (0 and 2), treatment (i.e., control and low and high irradiation), and their interaction, with replicate ID as a random effect. Second, we used a Generalized Additive Mixed Model with the Mgcv package in R (Wood et al., 2016) to examine the changes in sex ratio between transfer 2 and 20 because visual inspection of the data showed no simple relationship. The sex ratio was analyzed as a function of transfer (2, 5, 8, 11, 14, 17, and 20), treatment (i.e., control and low and high irradiation), and their interaction as fixed effects and replicate ID as a random effect. The smoothing was performed on the variable transfer in the function of treatment. A quasi‐binomial distribution and a logit link function were used and no overdispersion of data was observed. We analyzed the sex ratio in the reciprocal‐transplant experiment (data in Table S2), using a GLMM, with the environment of origin, environment of transplant, and the interaction between the environment of origin and environment of transplant as fixed effects. A binomial distribution and a logit link function were used and no overdispersion of data was observed. Replicate ID was included as a random effect.

## 3 RESULTS

### 3.1 Multigenerational experiment

**FIGURE 4:**
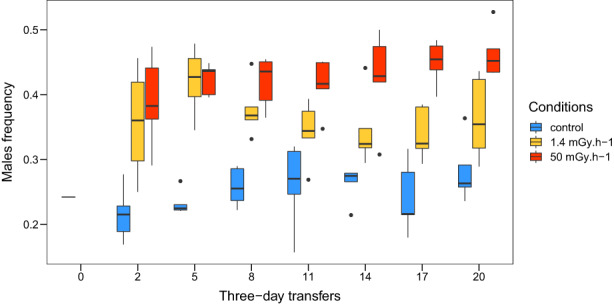
Boxplot of male frequency over time (i.e., 3‐day transfers: 0, 2, 5, 8, 11, 14, 17, and 20) for *C. elegans* populations living in different gamma radiation environments. Blue: Control; yellow: Low radiation (1.4 mGy.h^−1^); red: High radiation (50 mGy.h^−1^)

Between transfers 0 and 2, the significant transfer by treatment interaction, but non‐significant main terms, revealed an increase in male frequency in both irradiation conditions (1.4 and 50 mGy.h^−1^) but no change in the control treatment (Table 1; Figure 4).

**TABLE 1:** Effect of time (i.e., 3‐day transfer) and gamma irradiation condition (0, 1.4, and 50 mGy.h^−1^) on male frequency in *C. elegans*, between transfer 0 and 2

	Estimate	SE	*z* value	Pr(>|*z*|)
(Intercept)	−1.169	2.387e−01	−4.895	9.83e−07***
Time	−5.983e−02	1.332e−01	−0.449	0.653
Gamma low	6.297e−16	3.376e−01	0.000	1.000
Gamma high	1.479e−15	3.376e−01	0.000	1.000
Time:Gamma low	4.014e−01	1.881e−01	2.134	0.0328*
Time:Gamma high	3.850e−01	1.888e−01	2.039	0.0414*

*p < 0.05; **p < 0.01; ***p < 0.001.

Between transfers 2 and 20, the male frequency was estimated at 0.25, 0.37, and 0.43 for control, low irradiation treatment, and high irradiation treatment, respectively (Table 2; estimations shown have been transformed with the inverse‐logit function). Male frequency was higher in both the low and high irradiation treatments than in the control treatment (Table 2a; Figure 4). Male frequency decreased significantly across transfers in the low irradiation treatment and increased significantly in the control and high irradiation treatments (Table 2b; Figures 4 and S1).

**TABLE 2:** Effects of (a) irradiation condition (0.0, 1.4, and 50 mGy.h^−1^) and (b) time (EDF: Effective degrees of freedom) on *C. elegans* male frequency, between transfer 2 and 20

(a)	Estimate	SE	*t* value	Pr(>|*t*|)
(Intercept)	−1.077	0.03994	−26.97	<2e−16***
Gamma low	0.541	0.05403	10.01	<2e−16***
Gamma high	0.794	0.05504	14.43	<2e−16***

Approximate significance of smooth terms				
(b)	edf	Ref.df	*F*	*p*‐value
s(Time):Control	1	1	4.973	0.0280*
s(Time):Gamma low	1	1	5.162	0.0252*
s(Time):Gamma high	1	1	5.571	0.0202*

*p < 0.05; **p < 0.01; ***p < 0.001.

### 3.2 Reciprocal‐transplant experiments

**FIGURE 5:**
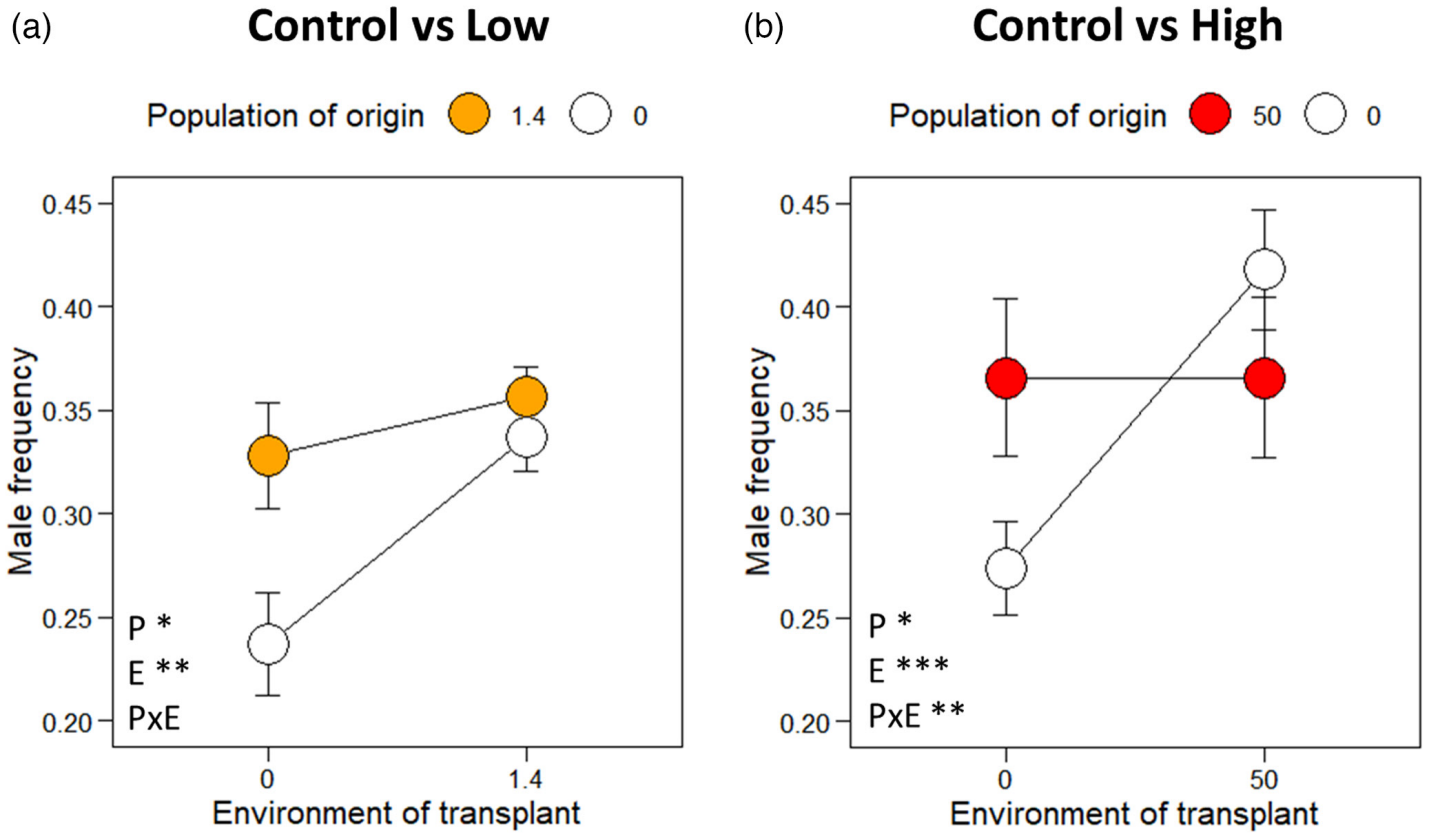
Sex ratio of *C. elegans* populations after four transfers of reciprocal transplant between control and both **(a)** low (a: 1.4 mGy.h^−1^) and **(b)** high (b: 50 mGy.h^−1^) radiation treatments. Dots represent the mean male frequency ± S.E for each new treatment. The color of the dot represents the populations' treatment during the multigenerational experiment. White: Control; yellow: Low radiation (1.4 mGy.h^−1^); red: High radiation (50 mGy.h^−1^). The significance of each main effect [population of origin (P), environment of transplant (e), and their interaction (P × E)] is indicated at the bottom left of each graph. *p < 0.05; **p < 0.01; ***p < 0.001

For transplants between the control and the low irradiation treatment, there was no significant interaction between the effects of the population of origin and the environment of transplant on male frequency (Table 3; Figure 5a). However, control populations transplanted into a low irradiation environment had significantly more males than low irradiation populations transplanted into a control environment (Table 3; Figure 5a).

**TABLE 3:** Results of generalized linear mixed models (GLMM) for male frequency in two reciprocal transplants between the environment of origin and the environment of transplant (between control and 1.4 mGy.h^−1^ and between control and 50 mGy.h^−1^)

Fixed effect	LR Chisq	Df	*p*
Control versus 1.4 mGy.h^−1^			
Population of origin	6.474	1	0.0109*
Environment of transplant	7.484	1	0.0062**
Population of origin: environment of transplant	2.601	1	0.1067
Control versus 50 mGy.h^−1^			
Population of origin	3.879	1	0.0489*
Environment of transplant	15.02	1	0.0001***
Population of origin: environment of transplant	6.782	1	0.0092**

*p < 0.05; **p < 0.01; ***p < 0.001.

Transplants between the control and the high irradiation treatment, in contrast, showed a significant interaction between the effects of the population of origin and the environment of transplant on male frequency (Table 3; Figure 5b). Male frequency increased in the control populations that were transplanted into the high irradiation treatment but did not change in the high irradiation populations that were transplanted into the control treatment.

### 3.3 SP model

**FIGURE 6:**
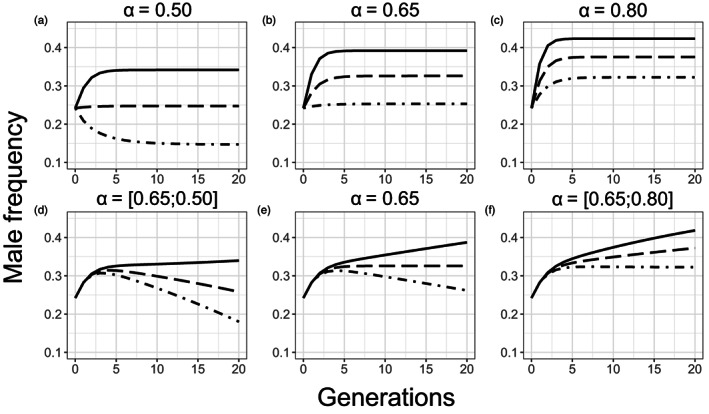
Model results showing changes in *C. elegans* male frequency over 20 generations as a function of male fertilization success (α) and the proportion of eggs not fertilized by males that are self‐fertilized (β). This model is adapted from Stewart and Phillips (2002). In (a), (b), and (c), α was set to 0.50, 0.65, and 0.80, respectively. The three lines correspond to three values of β: 0.10 (solid line), 0.15 (long dash line), and 0.20 (dot dash line). In (d), (e), and (f), β decreased from 0.15 to 0.10 (by −0.0025 per generation; solid line), β = 0.15 (long dash line), and β increases from 0.15 to 0.20 (by 0.0025 per generation) (dot dash line). In (d) α decreased from 0.65 to 0.50 (by −0.0075 per generation), in (e) α = 0.65 and in (f) α increases from 0.65 to 0.80 (by 0.0075 per generation)

The models showed that male frequency rapidly increased with male fertilization success (α: 0.50–0.80) and with a decrease in the proportion of eggs not fertilized by males that were self‐fertilized (β: 0.20–0.10; Figure 6a–c). Temporally stable values of α and β (Figure 6a–c) led to a plateau for male frequency after two to five generations. Increasing α or decreasing β across generations caused changes in sex ratio in the population like what we observed empirically (Figures 4 and 6f). The slight decrease in male frequency observed in the low irradiation treatment (Figure 4) was like the decrease in sex ratio caused by gradually decreasing α from 0.65 to 0.50 or increasing β between 0.15 and 0.20 (Figure 6d). The slow increase in male frequency observed in the high irradiation treatment (Figure 4) was like the results of the model when α was gradually increased from 0.65 to 0.80 or β was decreased from 0.15 to 0.10 (Figure 6f).

**FIGURE 7:**
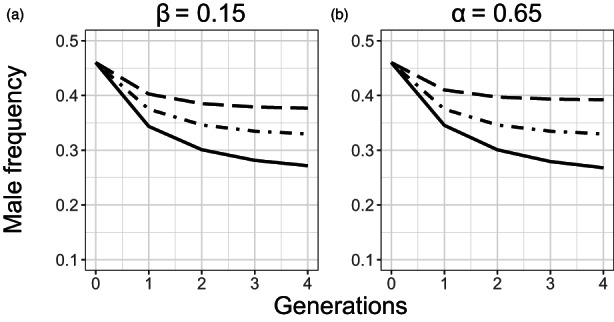
Model results showing the effects of *C. elegans* male fertilization success (α) and the proportion of eggs not fertilized by males that are self‐fertilized (β) on male frequency over four generations. This model is adapted from Stewart and Phillips (2002). (a) β = 0.15 and α = 0.52 (solid line), 0.65 (dot dash line), or 0.80 (long dash). (b) α = 0.65 and β = 0.20 (solid line), 0.15 (dot dash line), or 0.10 (long dash)

We then used the SP model to examine the effects of α and β in the reciprocal transplant experiment (Figure 7). Holding the values for male fertilization success (α) and the proportion of eggs not fertilized by males that are self‐fertilized (β) at those observed in the initial population (i.e., α = 0.52; β = 0.15; Figure 7a, solid line; or α = 0.65; β = 0.20; Figure 7b, solid line), produced a rapid decrease in male frequency from 0.46 to 0.27 in four generations. However, an increase in α (Figure 7a) or a decrease in β (Figure 7b) dampened this effect and better reproduced what was observed in the reciprocal transplant experiment. For example, for α = 0.80 and β = 0.15 (Figure 7a, long dash line), the male frequency at generation 4 was 0.38. Similarly, with α = 0.65 and β = 0.10 (Figure 7b, long dash line), the male frequency at generation 4 was 0.39.

**FIGURE 8:**
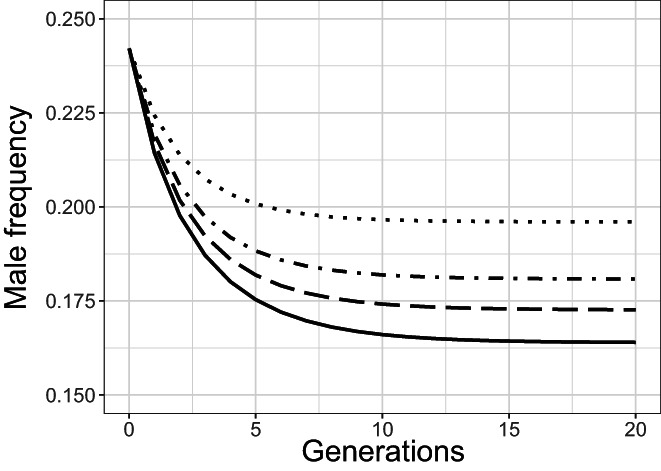
Models showing changes in male frequency over 20 generations in response to changes in the rate of nondisjunction of the X chromosome (*u*). This model is adapted from Stewart and Phillips (2002). The four lines correspond to four values of *u*: 0.005 (solid line), 0.0075 (long dash line), 0.010 (dot dash line), and 0.015 (dot line)

Finally, we used the SP model to examine the effects of the rate of nondisjunction of the X chromosome (*u*) on male frequency (Figure 8). The models showed that male frequency rapidly decreased and then reaches a plateau after 10 generations for all values of *u*. The level of the plateau increased with *u*. For example, the male frequency at generation 20 was 0.16 for *u* = 0.005 and 0.18 for *u* = 0.010 (Figure 8, solid line and dot dash line).

## 4 DISCUSSION

Our results show that ionizing radiation increased male frequency in *C. elegans* populations (Tables 1 and 2; Figure 4). Before irradiation, males made up slightly less than 25% of the populations. In the two irradiation treatment groups, the proportion of males increased by more than 10% after only two 3‐day transfers, and the increase was stronger under high than under low irradiation treatments. Over the subsequent 18 3‐day transfers, the male frequency increased slowly in the high irradiation treatment and decreased slowly in the low irradiation treatment, to reach 43% and 37% overall, respectively. In contrast, male frequency did not change significantly in the control treatment (Figure 4). The reciprocal‐transplant experiment showed that switching back to a non‐irradiated environment for four transfers did not reduce the high male frequency of the originally highly irradiated populations. Based on the SP model, these results may be explained by an increase in male fertilization success or a decrease in the proportion of self‐fertilized eggs under high gamma radiation conditions, that is, either irreversible or slow to reverse. These empirical and modeling results suggest that irradiation generates conditions that favor outcrossing in *C. elegans*, by providing a male fitness advantage over hermaphrodites.

### 4.1 Increased male frequency in response to radiation

Knowing that ionizing radiation has a deleterious impact on the reproduction of *C. elegans* (Buisset‐Goussen et al., 2014; Lecomte‐Pradines et al., 2017; Maremonti et al., 2019), we hypothesized that irradiation could directly influence the sex ratio. As expected, the male frequency increased in the presence of gamma radiation, and this effect was amplified under the higher dose rate (Table 1; Figures 4 and 5a). This effect is similar to that of other stressors, such as pesticide, starvation, or temperature, on male frequency in *C. elegans* (Lopes et al., 2008; Morran et al., 2009a; Rose & Baillie, 1979). Additionally, experimental tests investigating the influence of mutations on the evolution of outcrossing have shown an increase in male frequency in populations with a high mutation rate, induced by a genetic polymorphism or by the mutagen ethyl methanesulfonate (Cutter, 2005; Morran et al., 2009b). Male frequency also increases in response to stress in other species (e.g., *Pristionchus pacificus*, another Rhabditida, androdioecious species, under temperature stress Morgan et al., 2017, and *Daphnia magna* exposed to internal alpha irradiation, Alonzo et al., 2008). It thus seems that several environmental stressors lead to the same demographic changes in androdioecious species.

In *C. elegans*, increasing male frequency under ionizing radiation could have two, non‐exclusive, proximal causes: (1) an increase in outcrossing rate and (2) an increase in nondisjunction abnormality rate of the X chromosome. Results from our modified SP model support the first explanation (Figure 6). As previously described, self‐fertilization produces about 300 hermaphroditic offspring and is restricted by the quantity of sperm produced by hermaphrodites (Barr et al., 2018; Chasnov, 2013; Cutter et al., 2019). In contrast, outcrossing with several males can produce up to about 1000 offspring, with 50% of the offspring being male and 50% hermaphrodite (Chasnov, 2013; Cutter et al., 2019; Singson, 2001). In this configuration, hermaphroditic sperm could be the limiting gamete, and any further decrease in production could impact self‐fertilized offspring production. In fact, a decrease of about 30% in spermatid number and about 25% in self‐fertilized brood size has been observed at a dose rate of about 40 mGy h^−1^ (Buisset‐Goussen et al., 2014; Maremonti et al., 2019). Ionizing radiation can alter meiosis, decrease sperm activation and viability, and induce germ‐cell apoptosis. (Guédon et al., 2021; Maremonti et al., 2019). Under these conditions, if outcrossing persists, the decreased sperm quantity in hermaphrodites will create a fitness advantage for males, causing their frequency to increase. The model shows that increasing male frequency, in turn, increases the prevalence of outcrossing and positive feedback effects on male frequency. Outcrossing can also be favored if male *C. elegans* mate preferentially with older or sperm‐depleted hermaphrodites (Barr et al., 2018; Morsci et al., 2011). In fact, contact between mature hermaphroditic sperm and oocytes inhibits the production of sexual pheromones and limits attractiveness to males (Barr et al., 2018; Leighton et al., 2014; Morsci et al., 2011). Once their sperm is depleted, hermaphrodites produce more pheromone, stop avoiding males, and expel less seminal mass, all of which facilitate outcrossing (Chasnov, 2013). Since ionizing radiation reduces the number of their spermatids (Maremonti et al., 2019), it may cause hermaphrodites to become receptive to males earlier in their life.

The number of male *C. elegans* in the population may also increase because of nondisjunction abnormality of the X chromosome during meiosis (Broverman & Meneely, 1994). Usually, this step leads to hermaphrodites producing one XX and one Ø gamete, which produce a functional hermaphrodite and a male, respectively. The frequency of nondisjunction is modulated by genetic variation (Hodgkin et al., 1979; Mains et al., 1990; Teotónio et al., 2006) and increases under stressful conditions (e.g., high temperature in mice, Golbus, 1983, and *C. elegans*, Ayyadevara et al., 2014; Cutter et al., 2003 or gamma‐type radiation stress in *C. elegans*, Kim, 1985) and should, therefore, boost the production of males in irradiated populations (Kim, 1985; Rose & Baillie, 1979). Kim (1985) showed that an irradiation of 10 Gy doubled the nondisjunction of the X chromosome in *C. elegans*. However, the results of our SP model (Figure 8) suggest that the effect of the nondisjunction of the X chromosome on the frequency of males is rather small in our system. Doubling the frequency of the nondisjunction of the X chromosome increased the frequency of males by only 2%. It, thus, seems that non‐disjunction is not the main factor explaining the change in sex ratio in favor of males.

### 4.2 Evolutionary responses to ionizing radiation

After the second transfer of the multigenerational experiment, the male frequency increased slowly in the high irradiation treatment and decreased slowly in the low irradiation treatment. Using the modified SP model, we could reproduce similar trends by incorporating a gradual increase in male fertilization success or a gradual decrease in the proportion of self‐fertilization across transfers for the high irradiation treatment and conversely for the low irradiation treatment. These gradual changes probably reflect population evolution under stressful conditions and trait selection modifying male frequency. Additionally, populations transplanted from the high irradiation treatment to the control treatment maintained a high male frequency (Table 3; Figure 5). Results from the SP model suggest that this stability was caused by inertia in the level of outcrossing frequency in the population (Figure 7). These results suggest that even once the stressor stopped, males continued to have a fitness advantage over hermaphrodites for several generations. Such an advantage suggests that the selective pressures under high irradiation conditions may have caused genetic changes in both hermaphrodites and males that favored outcrossing over self‐fertilization. In agreement with the SP model, the slow decrease in male frequency under low irradiation treatment (Table 2; Figures 4 and S1) reflected a decrease in male fertilization success or an increase in the proportion of self‐fertilized eggs after many generations. These results could indicate that by adapting to ionizing radiation, outcrossing became less advantageous.

Alternatively, our results could reflect epigenetic effects on hermaphrodite reproduction or demographic inertia; the high proportion of males in the population leads to an unbalance in the reproductive success of both males and hermaphrodites, even generations after the stressor is removed. The high density of males could promote cross‐fertilization, maintaining a high male frequency (Cutter et al., 2003; Wegewitz et al., 2008). However, since the stressor is no longer present, self‐fertilization reproduction should become advantageous over outcrossing, leading to a progressive return to the original male frequency (Cutter et al., 2003; Wegewitz et al., 2008). In our reciprocal transplantation, this could explain the slight decrease observed in the frequency of males for the low irradiation treatment (Figure 5a). Given the relatively high male frequency in the high irradiation treatment before the reciprocal transplant experiment, it may have increased the inertia and lead to an even slower decrease in male frequency (Figure 5b). However, our modeling of reciprocal transplants (Figure 7) predicts a more rapid decrease in male frequency in the absence of population evolution than what we see in our experiment. This suggests that the inertia hypothesis alone cannot explain our results.

An increase in male frequency was also observed in *C. elegans* populations that evolved a resistance to levamisole, an anti‐parasitic pesticide (Lopes et al., 2008). The authors argued that outcrossing was advantageous under these conditions, and that the increase in male frequency was “an expression of the evolution of pesticide resistance” (Lopes et al., 2008). Other studies have indicated that male reproductive efficiency and the availability of hermaphrodites for outcrossing had a genetic basis (Gimond et al., 2019; Wegewitz et al., 2008). More generally, it has been strongly suggested that outcrossing is beneficial for fitness under stressful conditions and facilitates adaptation to stress (Morran et al., 2009a; Plesnar‐Bielak et al., 2017). Our results, however, suggest that a shift in the relative fitness of male and hermaphrodite reproductive strategies can explain the increase in male frequency, which, in turn, increases the amount of outcrossing. Rather than being the direct adaptive response of the population to novel stressors, outcrossing would be the by‐product of stress‐induced selection pressures on males and hermaphrodites and potential sexual conflict between them. This explanation has the advantage of not implying problematic group selection mechanisms (Grafen, 1984). The changes in male frequency observed in our experiments could also help us to understand the large disparity in male frequency among wild populations (Anderson et al., 2010; Sivasundar & Hey, 2005; Wegewitz et al., 2008). The relative proportion of males and hermaphrodites may have evolved under different stress regimes, with populations showing the highest rate of stressful events showing higher male frequencies than populations living under less stressful conditions.

## 5 CONCLUSION

Our results showed that ionizing radiation increased male frequency in a *C. elegans* population. This effect was amplified by increasing the dose rate. The results of our mathematical model suggest that the increase in the frequency of males could be explained by an increase in the fertilization success of males or a decrease in self‐fertilization. Moreover, after radiation was removed, males persisted at high proportions, as predicted by the mathematical model if an increase in the fertilization success of males or a decrease in the prevalence of self‐fertilization has taken place. This suggests that outcrossing in androdioecious populations may represent a demographic or evolutionary response to stress, such as exposure to ionizing radiation. These results show that ionizing radiation can cause prolonged changes in the reproductive strategy of a population, likely impacting its long‐term evolutionary history.

Our results highlight the importance of studying the evolutionary responses to pollution and the mechanisms involved for a robust assessment of the ecological risks of pollutants for populations. We strongly encourage the consideration of evolutionary parameters in the processes of ecological risk assessment of pollutants and more broadly of all anthropogenic stressors (climate change, etc.).

## CONFLICT OF INTEREST

The authors declare no conflict of interest.

## Supporting information


Figure S1

Table S1

Table S2

Appendix S1

## Data Availability

All data are in tables in supplementary files (Table S1 and S2).
