# Supplementary material for: Male frequency in Caenorhabditis elegans increases in response to chronic irradiation
Source: Evol Appl. 2022 Sep 2;15(9):1331–43. doi: 10.1111/eva.13420 (PMC9488675; doi:10.1111/eva.13420)
Supplement: Supplementary file 1 — Figure S1 [file EVA-15-1331-s004.pdf]

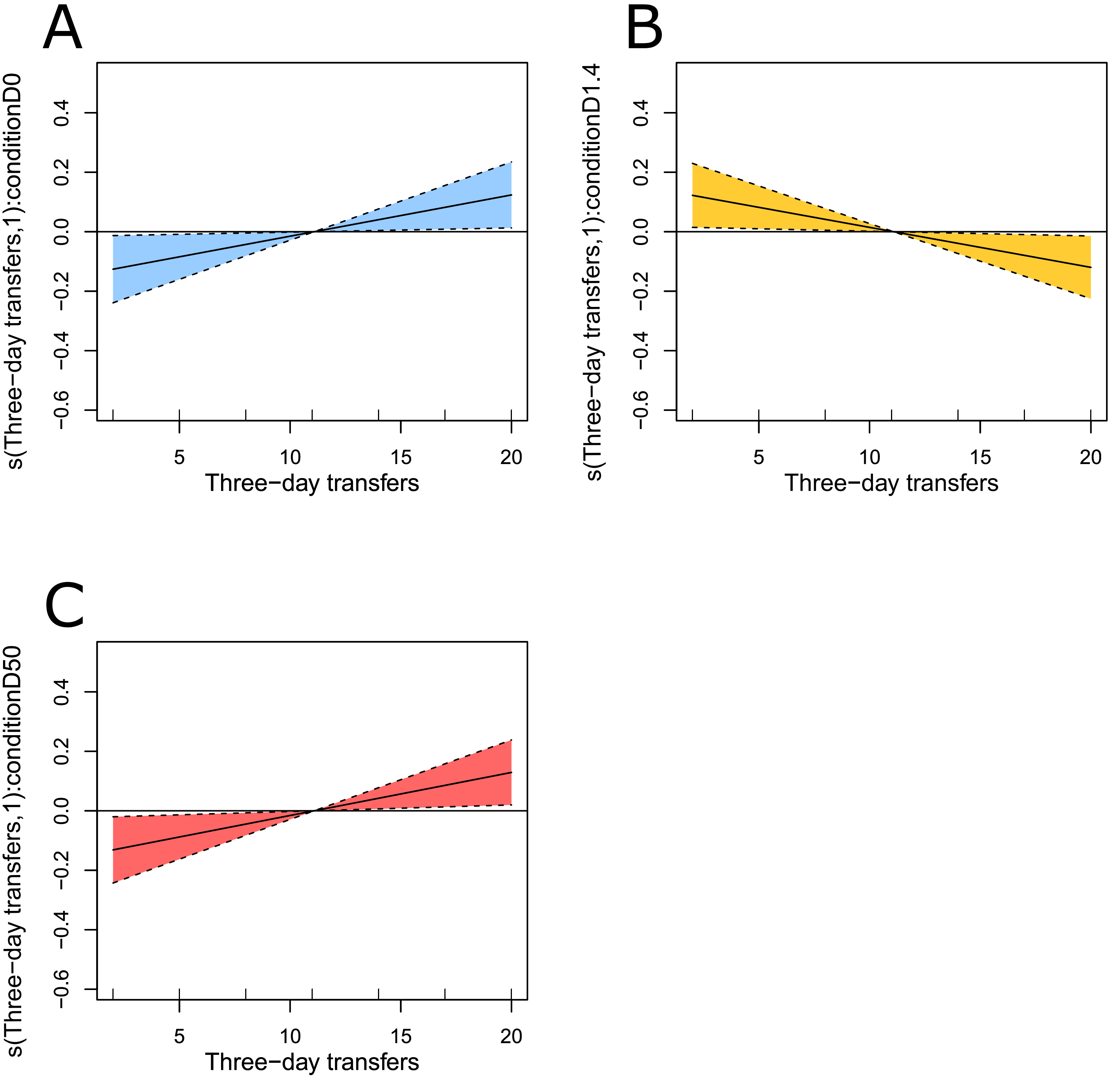
A
B
C
s(Three−day transfers,1):conditionD0
s(Three−day transfers,1):conditionD1.4
s(Three−day transfers,1):conditionD50
Three−day transfers
5
10
15
20
0.4
0.2
0.0
−0.2
−0.4
−0.6
