# Supplementary material for: Male frequency in Caenorhabditis elegans increases in response to chronic irradiation
Source: Evol Appl. 2022 Sep 2;15(9):1331–43. doi: 10.1111/eva.13420 (PMC9488675; doi:10.1111/eva.13420)
Supplement: Supplementary file 4 — Appendix S1 [file EVA-15-1331-s001.docx]

Figure S1 Partial response curves showing the relationship of the partial residuals of the response variable on the linear predictor scale and the relevant explanatory variables of the best approximate model. Plots were centered to have a mean value of zero along the y-axis, and the trends were used, rather than the actual values of the plots, to describe the responses to the smoothed explanatory variables. The graphical representations were estimated by GAMM. Plots show the partial effects of male frequency on time (3-day transfers) for **A.** Control **B.** 1.4 mGy.h^−1^ and **C.** 50 mGy.h^−1^. Shaded areas and dashed lines represent 95% confidence intervals.
